# Using dermal glucocorticoids to determine the effects of disease and environment on the critically endangered Wyoming toad

**DOI:** 10.1093/conphys/coab093

**Published:** 2021-12-23

**Authors:** Rachel M Santymire, Allison B Sacerdote-Velat, Andrew Gygli, Douglas A Keinath, Sinlan Poo, Kristin M Hinkson, Elizabeth M McKeag

**Affiliations:** 1 Davee Center for Epidemiology and Endocrinology, Lincoln Park Zoo, 2001 North Clark Street, Chicago, IL 60614, USA; 2 Department of Biology, Georgia State University, 100 Piedmont Avenue SE, Fourth floor, Atlanta, GA 30303, USA; 3 The Chicago Academy of Sciences, 2430 North Cannon Drive, Chicago, IL 60614, USA; 4 US Fish & Wildlife Service, Wyoming Ecological Services Field Office, 334 Parsley Boulevard, Cheyenne, WY, 82007, USA; 5 Department of Conservation & Research, Memphis Zoo, 2000 Prentiss Place, Memphis, TN 38112, USA; 6 USDA Forest Service, Nez Perce-Clearwater National Forests, 104 Airport Drive, Grangeville, ID 83530, USA; 7 Department of Biological Sciences, Arkansas State University, P.O. Box 599, State University, Jonesboro, AR 72467, USA

**Keywords:** ACTH, *Anaxyrus baxteri*, *Batrachochytrium dendrobatidis*, herpetofauna, stress

## Abstract

Amphibian populations are declining worldwide, and increased exposure to environmental stressors, including global climate change and pathogens like *Batrachochytrium dendrobatidis* (*Bd*), may be contributing to this decline. Our goal was to use a novel dermal swabbing method to measure glucocorticoid (GC) hormones and investigate the relationship among disease and environmental conditions in the critically endangered Wyoming toad (*Anaxyrus baxteri*). Our objectives were to (i) validate the use of dermal swabs to measure GCs using an adrenocorticotropic hormone (ACTH) challenge on eight captive toads (4 ACTH: 2 M, 2F and 4 saline as a control: 2 M, 2F), (ii) investigate stress physiology and disease status of toads across six reintroduction sites and (iii) compare dermal cortisol between reintroduced and captive toads. Dermal cortisol peaked immediately after the ACTH and saline injections. Faecal GC metabolites (FGMs) were significantly higher one week after the ACTH injection compared with the week before. Saline-injected toads had no change in FGM over time. Toads were only found in three reintroduction sites and dermal cortisol was similar across sites; however, reintroduced toads had higher dermal cortisol in August compared with June and compared with captive individuals. *Bd* status did not influence dermal cortisol concentrations. Dermal and faecal hormonal metabolite analyses can be used to study amphibian stress physiology and learn how environmental conditions are impacting population success.

## Introduction

Amphibian populations are declining worldwide (Stuart *et al.*, 2004; Wake and Vredenburg, 2008), and increased exposure to environmental stressors, such as global climate change and pathogens like *Batrachochytrium dendrobatidis* (*Bd*), has been proposed as one of several interactive causes of these widespread extirpations (Carey *et al.*, 1999; Wake and Vredenburg, 2008; Crawford *et al.*, 2012; Scheele *et al.*, 2019). Other environmental stressors, such as degraded habitats and environmental contaminants, have been shown to increase the secretion of glucocorticoids (GCs) in animals (Hopkins *et al.*, 1997, 1999; Homan *et al.*, 2003; Janin *et al.*, 2011).

GCs, including cortisol and corticosterone, are steroidal hormones, that are secreted in response to normal life history changes, such as metamorphosis and reproduction (Landys *et al.*, 2006; Crespi *et al.*, 2013). Their main purpose is to mobilize energy via gluconeogenesis (MacDougall-Shackleton *et al.*, 2019). GCs are also released in response to a threat, such as reduced food availability or extreme changes in climate, in an attempt to regain internal homeostasis (Romero and Wikelski, 2001; Romero, 2004; Carr, 2011). However, a prolonged elevation of GCs in an individual may lead to immunosuppression, behavioural changes, and/or reduced survival and reproduction (Saad *et al.*, 1987; Calow and Forbes, 1998; Bosson *et al.*, 2013; Cree *et al.*, 2003; Carr, 2011; Crespi *et al.*, 2013; Whirledge and Cidlowski, 2017). These effects can ultimately lead to population-level consequences or declines (Romero and Wikelski, 2001; Tracy *et al.*, 2006).

Along with other physiological and behavioural measures, GC profiles can be used as indices of site suitability by detecting differences in the acute and chronic GC productions of individual animals that inhabit different environments (Homyack, 2010). It is important to quantify baseline GC levels (circulating concentrations of GCs not excreted in response to a stressor), changes in GCs due to an acute stressor such as handling and the time it takes to recover with GC levels returning to baseline level to assess an animal’s full GC profile (Bliley and Woodley, 2012; Homan *et al.*, 2003). Many studies have found animals exposed to degraded habitats exhibit higher baseline GC levels or a depressed acute stress response, suggesting a dysfunctional hypothalamic–pituitary–adrenal (HPA) axis (Gendron *et al.*, 1997; Hopkins *et al.*, 1997, 1999; Homan *et al.*, 2003; Homyack, 2010; reviewed in Lucas *et al.*, 2006; Martínez-Mota *et al.*, 2007; Juster *et al.*, 2010). For example, common toads (*Bufo bufo*) from ponds in fragmented forest habitats had higher levels of urinary corticosterone, as well as lower body condition, than toads from ponds within more forested area (Janin *et al.*, 2011).

The immunosuppressive effect of prolonged stress may increase susceptibility to pathogens, such as the deadly chytrid fungus (*Bd*; Carey, 1993; McEwen, 1998; Marketon and Glaser, 2008; Kindermann *et al.*, 2012; Peterson *et al.*, 2013.; Rollins-Smith, 2017), in amphibians. Gabor *et al.* (2015) demonstrated an association between GCs and vulnerability to *Bd* infection in tadpoles and that GCs are affected by the aggressiveness of the infection. However, Hammond *et al.* (2020) found no relationship between *Bd* infection and salivary corticosterone in adults of two ranid species, and a context-dependent association in a third species in which *Bd*-infected individuals had lower baseline (field) and induced (captive) corticosterone than uninfected frogs, with captive frogs having greater corticosterone than wild frogs.

*Bd* has been identified as the main reason for the rapid decline of the critically endangered Wyoming toad (*Anaxyrus baxteri*; US Fish and Wildlife Service, 2015). The Wyoming toad is a small toad found only in Albany County, Wyoming, USA (Baxter and Stone, 1980). Historically, the toad mainly inhabited the floodplains and small ponds near the Laramie River, and populations were abundant until the 1970s (Baxter and Stone, 1980). While scientists originally speculated that pesticides, changes in agricultural practices and bacterial disease were responsible for the species’ decline (Baxter and Stone, 1980), it is now thought that *Bd* and loss of habitat due to changed hydrological regimes are the main reasons for the toad’s precipitous drop in numbers (US Fish and Wildlife Service, 2015). The Wyoming toad was listed as an endangered species in 1984 (US Department of the Interior, 1984), and the species is now considered extinct in the wild (IUCN, 2004).

The US Fish and Wildlife Service leads the effort to reintroduce captive-bred toads produced by the Wyoming Toad Species Survival Plan® (SSP) into six sites in Albany County (US Fish and Wildlife Service, 2018). Despite over 20 years of reintroduction efforts and recent successes such as documented breeding at multiple reintroduction sites, the reintroduced populations have not yet become self-sustaining (US Fish and Wildlife Service, 2018). Annual abundance estimates vary widely between sites, with some sites sustaining less than 10 toads a year, while surveyors frequently encounter dozens of toads at others. These site differences occur despite *Bd* being ubiquitous in Albany County and regardless of annual weather variation (US Fish and Wildlife Service, 2018). Reintroduction success is likely dependent on hitherto unknown environmental differences between sites.

While non-invasive faecal samples can be used to quantify GCs in larger wild animals, the secretive nature and small body size of amphibians makes the quantification of GCs more difficult. Fortunately, the *ex situ* breeding program for Wyoming toads allows us to study faecal GC metabolites. Faeces, when not deposited in water, can be an effective non-invasive method to study toad physiology. However, this is not a practical method for conducting field research. Previous field studies have used repeated blood draws or animal sacrifice (Hopkins *et al.*, 1999; Glennemeier and Denver, 2001; Narayan *et al.*, 2019); however, these invasive methods are not feasible to use in studies of the Wyoming toad because of its critically endangered conservation status. Therefore, our goals were to apply the results of previous research on American toads (*A. americanus*; Santymire *et al.*, 2018) to validate the use of dermal swabs for assessing GC levels in the Wyoming toad and compare the results to faecal GC metabolites (FGMs). Then, we applied this method to evaluate how *Bd* and the environment affect the Wyoming toad’s stress physiology.

Dermal swabs are ideal sample types because not all individuals will urinate or defecate upon handling. GC swabbing can occur concurrently as managers swab reintroduced toads for *Bd*. Dermal swabs can be quickly collected within 30 seconds, adding minimal handling time to the established sampling protocol. Recent work has used water sampling, where an amphibian is placed in a set amount of water for ~15 minutes up to several hours, thus allowing GCs to be secreted in the water (Reedy *et al.*, 2014; Gabor *et al.*, 2016; Forsburg *et al.*, 2019; Nagel *et al.*, 2019). However, it is difficult to determine if the GCs measured are in response to the animal’s environment or to being put in the container. This approach may be well suited for aquatic larvae, semi-aquatic or fully aquatic amphibians, but less so for terrestrial species such as toads. Another non-invasive sampling technique, which might be better applicable to field research, is measuring GCs in saliva (*Rana clamitans* and *R. pipiens*; Hammond *et al.*, 2018, 2020).

Our specific objectives were to (i) validate the use of the dermal swabbing method to measure GCs using an adrenocorticotropic hormone (ACTH) challenge, (ii) investigate the stress physiology of Wyoming toads across six different reintroduction sites and (iii) compare dermal GCs between reintroduced and captive Wyoming toads. We predicted that the non-invasive dermal swabbing method would accurately reflect the Wyoming toad’s stress physiology and that toads would exhibit higher GC concentrations at reintroduction sites with lower abundance estimates and higher prevalence of *Bd*.

## Material and methods

### Study animals and sites

**Figure 1:**
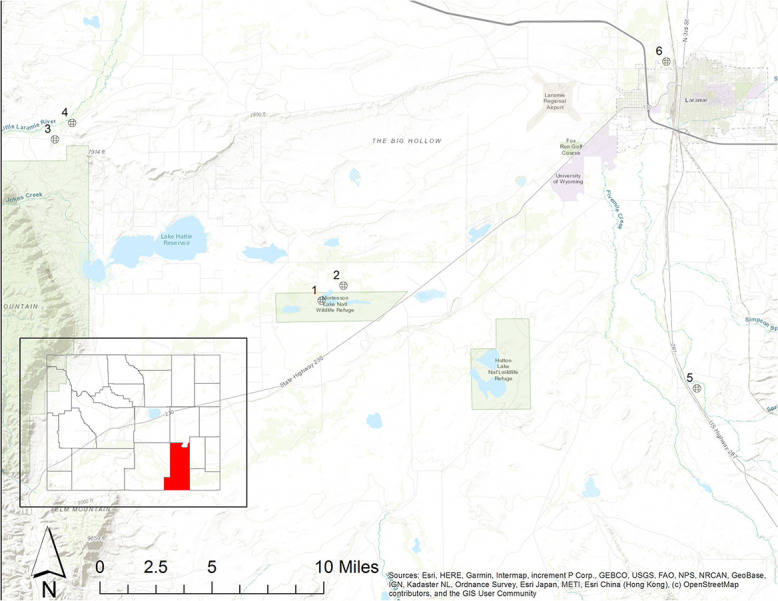
Six reintroduction sites where surveys for Wyoming toads were conducted: (1) Mortenson Lake National Wildlife Refuge (‘Mortenson’), (2) McGee, (3) Buford, (4) Lindzey, (5) Red Buttes and (6) Outrider. Toads were captured and sampled for GCs and chytrid fungus (*Bd*) using dermal swabs. All six sites were sampled in August, but only Mortenson was sampled in June. Inset map: David Benbennick, Public domain, via Wikimedia Commons.

All aspects of this research were approved by the Lincoln Park Zoo Research Committee and IACUC (2018-003), the Wyoming Toad Recovery Team, the US Fish and Wildlife Service (permit #TE704930-1) and the Wyoming Game and Fish Department (Chapter 33 permit #1025). The ACTH challenge experiment took place at Leadville National Fish Hatchery in Leadville, Colorado. Because of the endangered status of the species, we were permitted to use eight (four males, four females) captive Wyoming toads from the breeding program for the ACTH challenge. For reintroduced toads, we sampled individuals opportunistically in conjunction with USFWS annual population surveys. During these surveys, every adult toad encountered was captured, measured for mass and length, scanned for a uniquely identifying passive integrated transponder (PIT) tag and swabbed for *Bd* testing. In June 2018, Visual Encounter Surveys (VES) were conducted at Mortenson National Wildlife Refuge, a reintroduction site managed primarily for Wyoming toad recovery. In August 2018, VES were conducted over 2 weeks by teams in six reintroduction sites including Mortenson and five other sites (Buford, Lindzey, Red Buttes, Outrider and McGee) that are privately owned properties enrolled in Safe Harbor stewardship agreements with the local conservation district (Fig. 1). While a survey may take place over multiple days based on the size of the site, standardized survey blocks are only sampled once per survey. These VES occurred in conjunction with passive acoustic monitoring, which to date has been used to confirm toad presence, but not abundance, at each site. Mortenson, Buford, Red Buttes and McGee are characterized as dry shrub-steppe sites, with water provided by small ponds and lakes. Lindzey and Outrider are wetter sites with flowing water provided by the Little Laramie and Laramie Rivers and associated riparian vegetation. Both types of sites were historically occupied by Wyoming toads, but modern irrigation practices have changed the hydrological regime of the Laramie Basin and likely altered both types of systems (Peck and Lovvorn, 2001). Mortenson was the first Wyoming toad reintroduction site and has consistently retained the largest population of Wyoming toads (US Fish and Wildlife Service, 2015, 2018) while other sites have struggled to maintain their populations despite apparently similar habitat. While the riparian sites appear to have superior amphibian habitat, surveys have uncovered few toads, potentially due to low detectability in the thick riparian vegetation. During the August surveys, limited standing water was present at Red Buttes, Outrider and McGee. Distance and intervening habitat types prevent toads from dispersing between most of the reintroduction sites. It is possible individuals may move between Buford and Lindzey, although this activity has not been observed to date and a major road separates the two sites. It is highly likely that toads move between Mortenson and McGee as the two sites are adjacent without any obvious dispersal barriers.

### Validation of dermal swabs

We used an ACTH challenge to validate the use of GC dermal swab sampling. In amphibians, administering ACTH stimulates the interrenal glands to release GCs, which return to baseline within a few hours (Rollins-Smith, 2017). This methodology has been used in other amphibian species to validate non-invasive measurements of GC production from dermal swabs, urine, saliva, water and faeces (Table 1).

**Table 1:** Amphibian species and non-invasive GC sampling techniques in which ACTH challenges have been used to examine stress response

Species	Sample type	GC	Citation
Green treefrog (*Hyla cinerea*)	Dermal	Cortisol	Santymire *et al.* (2018)
Eastern newt (*Notopthalamus viridescens*)	Dermal	Cortisol	Santymire *et al.* (2018)
American toad (*Anaxyrus americanus*)	Dermal	Cortisol	Santymire et al. (2018)
Edible bullfrog (*Pyxicephalus edulis*)	Dermal	Cortisol	Scheun *et al.* (2019)
Harlequin frog (*Atelopus* spp.)	Faeces	Corticosterone	Cikanek *et al.* (2014)
American bullfrog (*Rana catesbeiana*)	Saliva	Corticosterone	Hammond *et al.* (2018, 2020)
Green frog (*Rana clamitans*)	Saliva	Corticosterone	Hammond *et al.* (2018, 2020)
Northern leopard frog (*Rana pipiens*)	Saliva	Corticosterone	Hammond *et al.* (2018)
Wood frog (*Rana sylvatica*)	Saliva	Corticosterone	Hammond *et al.* (2020)
Bell frog (*Litoria raniformis*)	Urine	Corticosterone	Germano *et al.* (2009)
Fijian ground frog (*Platymantis vitiana*)	Urine	Corticosterone	Narayan *et al.* (2010)
Stoney Creek frog (*Litoria wilcoxii*)	Urine	Corticosterone	Kindermann *et al.* (2012)
Australian Great Barred frogs (*Mixophyes fasciolatus*)	Urine	Corticosterone	Graham *et al.* (2013)
Maud Island frogs (*Leiopelma pakeka*)	Urine	Corticosterone	Germano *et al.* (2012)
Cane toads (*Rhinella marina*)	Urine	Corticosterone	Narayan and Hero (2014)
San Marcos salamander (*Eurycea nana*)	Water	Corticosterone	Gabor *et al.* (2016)
Eastern newts (*Notophthalmus viridescens*)	Water	Corticosterone	Reedy *et al.* (2014)
Rio Grande leopard frogs (*Rana berlandieri*)	Water	Corticosterone	Forsburg *et al.* (2019)
Mudpuppy (*Necturus maculosus*)	Water	Corticosterone	Nagel *et al.* (2019)

To control for effects from seasonal and daily GC cycles, we conducted the ACTH challenge between 10 am and 4 pm, when baseline levels are steady, and in May, before the toad’s breeding season begins in early June and cortisol tends to peak (Pancak and Taylor, 1983). Since moving an animal into a new enclosure can elicit an acute stress response (Bosson *et al.*, 2013; Letty *et al.*, 2000), toads used for the ACTH challenge experiment were individually housed in tanks for at least 2 weeks prior to the experiment to allow their GC levels to stabilize. This allowed us to collect opportunistic faecal samples from individual toads as another means of GC analysis (Schwarzenberger, 2007). Every faecal sample that was not contaminated with water or urine was collected for 2 weeks before and after the ACTH experiment. Samples were stored in a −20°C freezer until they were shipped to the Davee Center Endocrinology Laboratory at the Lincoln Park Zoo.

Wearing powder-free, latex gloves, with fresh gloves for each sample collected, each toad was picked up, held and immediately swabbed six times dorsally, spanning 2.54 cm with a sterile cotton-tipped plastic swab (Puritan Cotton-Tipped Applicators, #VWR 10806-005). Swabs were placed in individually labelled 2.0-mL tubes containing 1 mL of 70% ethanol. This served as a baseline sample of GC levels. Then, the toad was intraperitoneally injected with either ACTH (Cortrosyn, Amphastar Pharmaceuticals, Rancho Cucamonga, CA, 6959006H/9-10; *n* = 4 toads; two males, two females) or a saline control (*n* = 4 toads; two males, two females). ACTH was administered at 2.5 uL/g body mass, which equates to 0.446 μg ACTH/g bodyweight in 100-μL saline vehicle (0.9% NaCL; Narayan *et al.*, 2010; Santymire *et al.*, 2018), and saline was administered at 100 μL 0.9% NaCL to act as a control. Following the injection, we immediately collected a second swab and then repeated the swab collection every 15 minutes for 1 hour followed by an additional hour where we collected samples every 30 minutes. This protocol resulted in a total of eight swabs: baseline, immediately following the injection, 15, 30, 45, 60, 90 and 120 minutes post-injection. We stored all swabs at 5°C until shipping to Lincoln Park Zoo’s Endocrinology Lab.

### Sample processing and hormonal analysis

Swabs were processed using previously described methods (Santymire *et al.*, 2018). Briefly, sample vials containing the plastic swab in 1 mL of ethanol were shaken on a mixer (Glas-col, Terre Haute, IN, USA; setting 60–70 rpm) for 5 minutes. Then, 500 μL of 70% ethanol was pipetted into new test tubes. We used forced air in a warm water bath at 60°C to evaporate the ethanol and reconstituted the samples with 500 μL of phosphate buffered saline (PBS; 0.2 M NaH_2_PO_4,_ 0.2 M Na_2_HPO_4_, NaCl). Two to three glass beads were added to each tube. Then, they were vortexed briefly and sonicated for 20 minutes. Samples were then shaken again on the Glas-col mixer (60–70 rpm) for 30 minutes and analysed on cortisol and corticosterone enzyme immunoassays (EIAs).

Faecal samples were analysed using previously described methods (Poessel *et al.*, 2011). Briefly, samples were lyophilized and crushed. Then, 90% ethanol was added to 0.02 g dried faeces and mixed for 30 minutes on the Glas-col mixer (setting 60–70 rpm). Samples were centrifuged for 20 minutes and supernatant was poured off. Extracts were dried under forced air in a water bath at 60°C. Similar to the swabs, faecal extracts were reconstituted in PBS buffer, sonicated for 20 minutes and mixed for 30 minutes. Samples were analysed on both cortisol and corticosterone EIAs to determine the appropriate GCs to measure Wyoming toad stress physiology.

For the cortisol EIA, the polyclonal antiserum and horseradish peroxidase (HRP) ligands (R4866; provided by C. Munro, Davis, CA) were used at 1:375000 and 1:200000. Cross-reactivity to the cortisol antiserum has been previously published (Munro and Stabenfeldt, 1984; Young *et al.*, 2004; Loeding *et al.*, 2011). The cortisol EIA was biochemically validated in the laboratory by demonstrating the parallelism between binding inhibition curves of sample hormones (2× concentrated to 1:8) and the cortisol standard (swab, *r* = 0.989; faeces, *r* = 0.999; Supplementary Fig. S1A and C); and the significant recovery (≥90%) of exogenous cortisol (1.95–500 pg/well) added to pooled swab samples (swab, y = 1.01x—1.39; R^2^ = 0.999; *P* < 0.001; faeces, y = 1.03x—2.45; R^2^ = 0.999; *P* < 0.001; Supplementary Fig. S2A and C). Assay sensitivity was 1.95 pg/well. The intra-assay coefficient of variation (CV) was < 10% and the inter-assay CV was <7%.

For the corticosterone EIA, polyclonal corticosterone antiserum (CJM006) and (HRP) (provided by C. Munro, University of California, Davis, California) were used at dilution rates of 1:225000 and 1:200000, respectively. The cross-reactivity for this corticosterone antibody has been published previously (Santymire and Armstrong, 2010). The corticosterone EIA was validated biochemically by demonstrating the parallelism between binding inhibition curves of sample hormones (2× concentrated to 1:8) and the corticosterone standard (swab, *r* = 0.980; faeces, *r* = 0.995; Supplementary Fig. S1B and D); and the significant recovery (≥90%) of exogenous corticosterone (1.95–500 pg/well) added to pooled samples (swab, y = 0.84x + 3.19, R^2^ = 0.9997, *P* < 0.001; faeces, y = 0.986x—1.177; R^2^ = 0.998; *P* < 0.001; Supplementary Fig. S2B and D). Assay sensitivity was 1.95 pg/well. The intra-assay CV was < 10% and the inter-assay CV was <7%.

### Field sampling reintroduced Wyoming toads

Given the critically endangered status of Wyoming toads, managers seek to minimize the handling time for individual toads in field sampling protocols. We collected samples from toads found during surveys at the Mortenson reintroduction site in June that were being conducted for another research project and from all toads encountered during routine annual monitoring surveys of six reintroduction sites in August. The toads sampled in June were potentially in breeding status, unlike the captive toads sampled in May or the August surveys. While the breeding status of the sampled toads had the potential to affect the study’s results, minimizing the handling and repeated sampling of individuals was important due to the species’ conservation status. Therefore, GC dermal swabbing was conducted simultaneously with VES. Upon capture, each toad was immediately swabbed twice before any other measurements were taken. First, using the GC dermal swabbing method on the dorsum for a baseline GC sample, and second, to test for the presence of *Bd*. *Bd* swab collection followed the standard USFWS protocol, swabbing the venter and undersides of the limbs and feet 20–30 times total (Doug Keinath, USFWS, pers. comm.). Because the GC swab was collected from the dorsum, it should not impact *Bd* zoospore detection from the venter of the toads. Fresh powder-free latex gloves were used for sample collection for each toad encountered. The *Bd* swabs were stored dry in tubes and analysed using polymerase chain reaction at the US Fish and Wildlife Service’s Bozeman Fish Health Center in Bozeman, Montana. All GC swabs were stored at 5°C until they were shipped to Lincoln Park Zoo for processing. For all days of surveying, weather data were downloaded from https://www.wunderground.com to test for the potentially confounding effects of weather on GC levels.

### Data analysis

To determine the GC that was the most biologically relevant for both the swab and faecal results, we graphed both cortisol and corticosterone concentrations for the ACTH challenge samples and then compared them using Pearson correlation using Sigma Plot (version 12). Then, to examine the effects of the ACTH or saline on both GCs production, we used the absolute change [i.e. the fold increase (Romero, 2004)] by calculating the quotient between the pre-stress samples and samples post-injection. We averaged the fold-change by individual and conducted a paired t-test to determine if the results between the two GCs were different. We evaluated both GCs visually looking for expected responses to the injections. After determining the GC that best represented the stimulation of the hypothalamus–pituitary–interrenal (HPI) axis via the ACTH challenge, we tested the dermal GC and FGM data for normality using Shapiro–Wilk for normality assumption testing and Levene’s median test for equal variance assumption. Then, all GC values (both swab and faecal) were log-transformed. Although we had only four males and four females, we wanted to ensure that sex was not influencing GC production before grouping them for further analyses. Therefore, we used the pre-injection sample and used a *t*-test or a Mann–Whitney Rank Sum test if data were not normal to determine if GCs (both swab and faecal) differed between the sexes, We considered any time point that was at or above 1.5-fold higher to be elevated and indicating a response to the stressor (Santymire *et al.*, 2018). To determine if fold increase in dermal GC differed between time (0 to 120 minutes post-injection) and treatment (ACTH vs. saline), we used a two-way repeated measures ANOVA with a Student–Newman–Keuls *post hoc* analysis. Unlike the timed swab sampling, the toads did not defecate on the same days. Therefore, to compare the response (via fold-change) to the treatment, we averaged the samples 1 week before the injection (either saline or ACTH) and compared them with the average of samples defecated 1 week after the injection using a t-test (or Mann–Whitney Rank Sum Test if data were not normal) since there was only one value for pre- and post-injection per individual.

For reintroduced toads, we used a one-way ANOVA (or Kruskal–Wallis ANOVA on Ranks with a Dunn’s *post hoc* analysis if transformed data were not normal) to compare dermal GCs among males, females and juveniles with unknown sex. We then used a two-way ANOVA to compare dermal GC concentrations between sites and month sampled with a Student–Newman–Keuls *post hoc* analysis. We used the same analysis to determine the effects of *Bd* status on GC production. Finally, we used a one-way ANOVA with a Student–Newman–Keuls *post hoc* analysis (or Kruskal–Wallis ANOVA on Ranks with a Dunn’s *post hoc* analysis if transformed data were not normal) to compare reintroduced toads by month and captive toad dermal GCs, Values are presented as mean ± SE. For all analyses, *P* < 0.05 was considered significant.

## Results

### Determining the appropriate GC to measure HPI stimulation

Dermal cortisol was positively correlated (*r* = 0.252; *P* = 0.04) with corticosterone in the dermal GCs. Additionally, the fold-change response was similar (t_7_ = −0.204; *P* = 0.844) between both GCs. While we found similar hormonal profiles when comparing cortisol and corticosterone, two individuals had different responses to the saline and the ACTH. Female #138695 had an elevated (≥1.5-fold) response in seven out of eight samples after the saline injection for corticosterone but only had an elevated (2.2-fold) increase at 0 min post-injection with the cortisol results (Supplementary Fig. S3). Female # 139170 had an average of a 3.4-fold increase after the ACTH injection when using the cortisol EIA but had no response (mean, 0.5-fold change) when we used the corticosterone EIA (Supplementary Fig. S4). Therefore, we choose to continue the analysis using only the cortisol results.

For FGMs, faecal corticosterone and cortisol metabolites were positively correlated (*r* = 0.784; *P* < 0.001). The responses were similar (W = 47.0; *P* = 0.153) between the two GCs, but two individuals had different results. For Male #139008, the cortisol EIA found an increase (7.6- and 2.5-fold) at Days 2 and 4 post-injection, but the corticosterone EIA did not (1.7- and 1.3-fold increase, respectively; Supplementary Fig. S5A). For Male #139131, the corticosterone EIA found an increase (3.3-fold) post-saline injection, but the cortisol EIA did not (1.1-fold increase; Supplementary Fig. S5B). Because of these differences, we choose to use faecal cortisol metabolite results.

**Figure 2:**
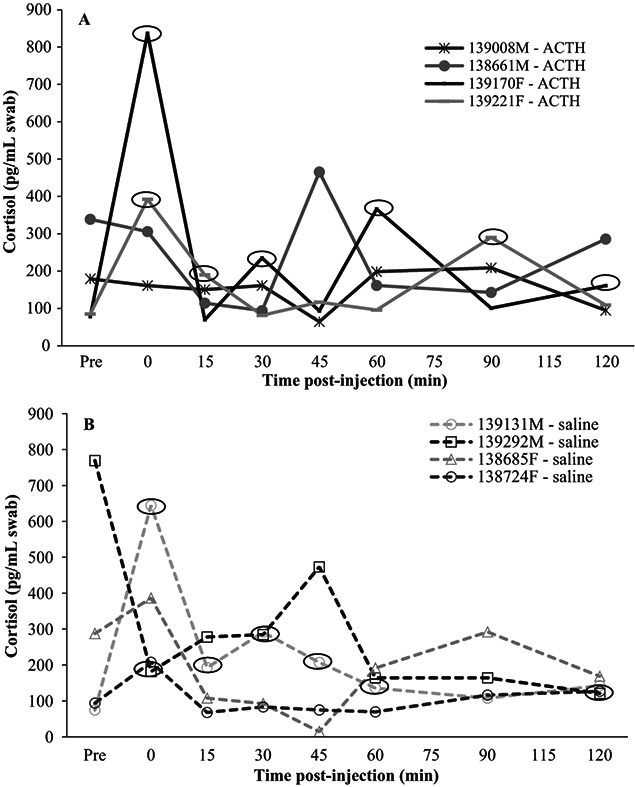
Dermal cortisol (pg/ml swab) results over time in captive Wyoming toads after receiving [A] ACTH (two males, two females) and [B] saline (two males, two females). Black circles indicate elevated samples (≥1.5 fold higher than pre-injection sample).

### Validation of GC analysis

**Figure 3:**
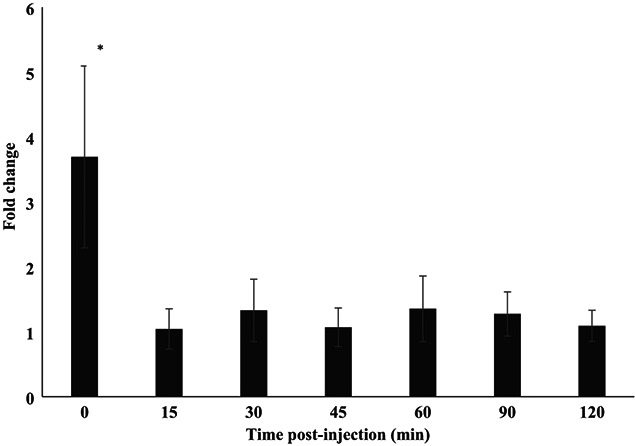
Mean (±SE) fold change (Romero, 2004) of dermal cortisol (pg/ml swab) from pre-injection sample in captive Wyoming toads after receiving ACTH (two males, two females) or saline (two males, two females). Asterisks indicate differences (*P* < 0.05) over time.

When using the pre-injection sample only, captive males (*n* = 4) and females (*n* = 4) had similar (t_6_ = 0.892; *P* = 0.407) dermal cortisol values (Fig. 2A and B); therefore, we combined the results from both sexes in further analyses. When comparing the dermal cortisol responses to the treatment, treatment did not influence (F_1,36_ = 0.201; *P* = 0.669) dermal cortisol; however, time affected (F_6,36_ = 3.32; *P* = 0.011) concentrations. Specifically, Time 0 pre-injection had the highest (*P* < 0.05) fold-change response compared with all other time periods (Fig. 3). Saline resulted in one elevated swab (2.2-fold increase) for Female #138724 at 0 minute, but then her cortisol returned to baseline. Female #139221 had an immediate increase (4.6-fold at 0 minute, 2.2-fold at 15 minutes) post-ACTH, but then returned to baseline at 30 minutes. She did have another increase (3.0-fold) at 90 minutes but started to decrease (1.3-fold) at 120 minutes. Female #139170 had an immediate response (10.7-fold at 0 minute), but then returned to baseline at 15 minutes. She had three more peaks (3.0-fold at 30 minutes, 4.7-fold at 60 minutes and 2.1-fold at 120 minutes) but returned to near baseline (1.2-and 1.3-fold higher than baseline) in between the peaks (Fig. 2A and B). Male #139131 had nearly all elevated samples (8.6-fold at 0 minute, 2.6-fold at 15 minutes, 3.9-fold at 30 minutes, 2.8-fold at 45 minutes, 1.8-fold at 60 minutes and 1.9-fold at 120 minutes) post-saline injection and never returned to baseline. Male # 139292 never responded to the saline. Additionally, both ACTH-injected males (#139008 and #138661) had no elevated peaks (Fig. 2A and B).

**Figure 4:**
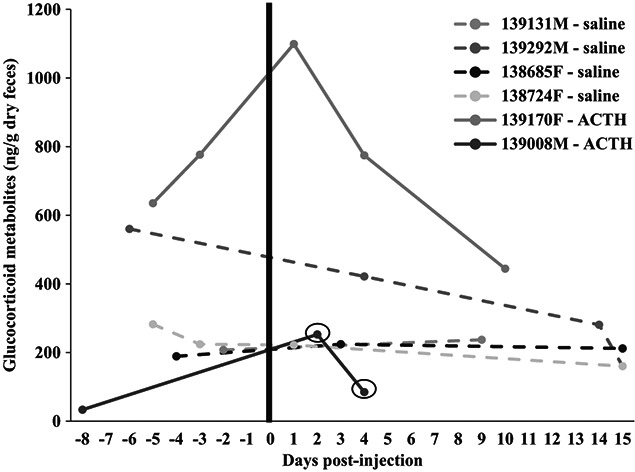
FGM (ng/g dry faeces) results over time in captive Wyoming toads [two ACTH (one male, one female); four saline (two males, two females)]. Solid lines are individuals that were injected with ACTH. Dash lines are the saline-injected individuals. Black circles indicate elevated samples (≥1.5 fold higher than pre-injection sample).

For FGMs, four to five faecal samples were collected from each toad over the 1 month of sample collection. Unfortunately, following the ACTH injection, two of the four toads (one male and one female) defecated repeatedly in water, and we were unable to collect an uncontaminated sample. When using the pre-injection samples only, captive males and females had similar (t_4_ = −0.76; *P* = 0.490) FGM values (Fig. 4). While the treatment (saline vs. ACTH; F_1,4_ = 0.50; *P* = 0.52) and the timeframe (the week before vs. week after the injection; F_1,4_ = 5.45; *P* = 0.08) did not influence FGMs, there was an interaction between week and treatment (F_1,4_ = 9.71; *P* = 0.036) with higher (q = 4.72; *P* = 0.029) FGMs found the week after the ACTH injection (552.8 ± 336.3.0 ng/g) compared with the week before (369.6 ± 336.3 ng/g). Treatment did not influence (U = 0.0; *P* = 0.133) fold-change responses in toads. Specifically, none of the four saline toads had elevated samples post-injection; however, the ACTH male that produced uncontaminated faecal samples had two elevated samples (7.6-fold at 2 days and 2.5 at 4 days) post-ACTH. We did not capture when his FGMs returned to baseline (i.e. no useable samples after Day 4). One female had a nearly elevated peak (1.4-fold higher than pre-injection FGM) peak 1 day post-ACTH injection but had returned to baseline by Day 4 (Fig. 4).

### Reintroduced Wyoming toads

**Figure 5:**
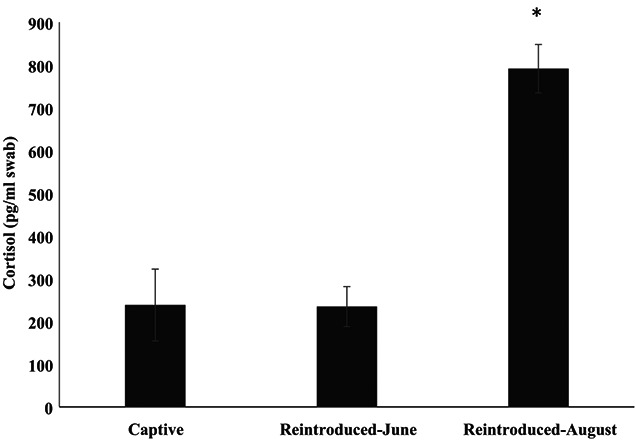
Mean (± SEM) dermal cortisol (pg/ml swab) results in Wyoming toads sampled at a captive facility (*n* = 8; using pre-injection sample only) and at the Mortenson reintroduction site in June (*n* = 6) and at the three reintroductions (Buford, Lindzey and Mortenson) in August (*n* = 27). Asterisks indicates a difference (*P* < 0.001) between months and location.

No Wyoming toads were found at the McGee, Red Buttes or Outrider sites. Six males were sampled at Mortenson in June. Mean temperature was 62.4°F (mean range, 50 to 73°F) with a total of 1.09 inches of precipitation for the entire month. In August, 12 (6 females, 6 males) reintroduced toads (one with PIT tag) were sampled at the Mortenson site. At the Buford site, 10 (2 females, 2 males, 6 unknown) reintroduced toads (2 PIT tags) were sampled. Finally, at the Lindzey site, five toads (all females) were swabbed. Mean temperature was 63.5°F (mean range, 51 to 72°F) with a total of 0.73 inches of precipitation for the entire month. Overall, reintroduced females (712.8 ± 68.0 pg/mL swab) had similar (H_2_ = 0.22; *P* = 0.898) dermal cortisol to males (972.0 ± 101.0 pg/mL swab) and juveniles of unknown sex (679.8 ± 109.9 pg/mL swab); therefore, we grouped them for further analyses. Dermal cortisol (708.0 ± 59.5 pg/mL swab) was similar (F_2,28_ = 0.029 *P* = 0.972) among the three sites. However, month affected (F_1,28_ = 28.97; *P* < 0.001) dermal cortisol concentrations with dermal cortisol three-times higher (q = 7.24; *P* < 0.001) in August (791.4 ± 56.8 pg/mL swab) compared with June (257.7 ± 49.8 pg/mL swab; Fig. 5).

*Bd* was present at all three sites where toads were sampled. Two of the six male Wyoming toads sampled in June at the Mortenson were *Bd*+; however, in August five of six males and four of six females were *Bd*+. Two of the five females at the Lindzey site were *Bd*+. Finally, all 10 Wyoming toads found at the Buford site were *Bd*+. Reintroduced toads that were *Bd* + (*n* = 23, 770.4 ± 69.9 pg/mL swab) had similar (F_1,28_ = 0.086; *P* = 0.772) dermal cortisol to *Bd*-toads (n = 9, 467.1 ± 100.0 pg/mL swab).

### Comparing reintroduced to captive Wyoming toad

Location (captive vs. reintroduction site) influenced (H_2_ = 22.13; *P* < 0.001) dermal cortisol. Specifically, reintroduced toads sampled in August (*n* = 27; 791.4 ± 56.8 pg/mL swab) had higher (Q = 3.92; *P* < 0.05) dermal cortisol than captive toads (*n* = 8; pre-injection sample only, 238.3 ± 83.9 pg/mL swab) and reintroduced toads (Q = 3.01; *P* < 0.05) sampled in June (*n* = 6; 257.7 ± 49.8 pg/mL swab), which were similar (Q = 0.20; *P* > 0.05) to each other (Fig. 5).

## Discussion

Understanding how environmental conditions are impacting endangered species is vital to their conservation and management. By monitoring wildlife stress physiology, we can gain some insight into how they are coping with these conditions, and in turn, better inform our conservation decisions, such as choice of release sites for reintroduction. Several methods have been employed to monitor GCs in wildlife, including blood, faeces, urine, feathers, water, saliva and hair (Madliger *et al.*, 2018; Palme, 2019). However, it is more challenging when the species is small, secretive and does not have hair, like amphibians. A recent study (Santymire *et al.*, 2018) validated the use of dermal swabs to study amphibian stress physiology. Here, we validated this technique along with FGM analysis for the Wyoming toad. We used an ACTH challenge in captive Wyoming toads to ensure that the cortisol that we measured was biologically relevant to the toad’s physiology. In vertebrates, administering ACTH stimulates the adrenal glands (interrenals in amphibians; Rollins-Smith, 2017) to release GCs, which will return to normal values within a few hours (Touma and Palme, 2005). An ACTH challenge has been used to validate noninvasive GC sampling techniques in 17 amphibian species using dermal swabs, urine, saliva, faeces and water (Table 1). Here, GC increases due to the ACTH injection and measured via dermal cortisol, varied. However, our results are limited because of our small sample size (four individuals per treatment group), which was related to this species being endangered.

### Cortisol vs. corticosterone

Our first goal was to use the ACTH challenge to determine the most appropriate GC to measure HPI stimulation for Wyoming toads. This is important to ensure that the analysis is an accurate reflection of a species’ ability to cope with its environment (Palme, 2005). This can be challenging because cortisol and corticosterone are produced using similar pathways and enzymes; therefore, both may be produced in response to similar or different stimuli and may be produced at different magnitudes (Koren *et al.*, 2012). Koren *et al.* (2012) found a positive association between cortisol and corticosterone in 9 of 13 species that excreted cortisol as the predominant GC. Here, we found that dermal cortisol and corticosterone had similar responses to saline and ACTH (via fold-change) with the exception of two (out of eight) individuals. For FGM analysis, cortisol and corticosterone results were also highly correlated. We found a major difference between the two hormone analyses in one individual (139008); this male received ACTH and had higher fold increases using the cortisol EIA than when using the corticosterone. Then, the response to saline in one female (139131) was elevated when using the corticosterone EIA vs. cortisol EIA (3.3- vs. 1.1-fold increase). We chose to use the cortisol EIA results for the FGM analysis knowing that the functionality of each GC may vary during different age classes (Palme, 2005), circannual cycles (Boswell *et al.*, 1994), metabolic effects (Devenport *et al.*, 1989) and affinity to binding to corticosteroid-binding globulin during transportation around the body via blood (Devenport *et al.*, 1989; Kenagy and Place, 2000). These types of analyses would include examining blood levels and potentially binding ability to receptors and would require more invasive analyses that cannot be done on endangered species.

### Dermal cortisol responses

The response time between a stressor and dermal hormone release is suggested to be similar to blood or saliva (Santymire *et al.*, 2018). GC production in response to a stressor may be as rapid as 3 minutes, especially in smaller species (Romero and Reed, 2005). Previously, ACTH injections have elicited increases in plasma cortisol <1 hour in the European frog (*R. esculenta*; Jurani *et al.*, 1973). Here, we observed immediate increases in cortisol post-injection (of either ACTH or saline) in four of the eight individuals. Most individuals returned to dermal cortisol baseline with 15–30 minutes after the peak.

With a limited sample size of four from each sex, we found that captive male and female Wyoming toads had similar dermal cortisol concentrations. The lack of influence of sex on dermal corticosterone was observed in the edible bullfrog (Scheun *et al.*, 2019). However, female Wyoming toads may be more responsive to stressors as they produced higher peaks than males (mean of 3-fold higher compared to less than 1-fold higher in males). These responses were similar to the response of an American toad in a previous study, which had dermal cortisol peaks at 0, 15, 45, 60, 90, and 120 minutes post-ACTH (Santymire *et al.*, 2018). However, in the edible bullfrog, the peak dermal corticosterone in response to an ACTH injection varied between 45 minutes and 10 hours (Scheun *et al.*, 2019).

The challenge and a limitation with using an ACTH injection to validate dermal cortisol is that the toads must be handled every time they are swabbed, which may cause repeated episodes of stress and peaks in dermal cortisol (Santymire *et al.*, 2018). This might explain why there were immediate and continuing responses throughout the 2 hours of sampling for the females. It also might explain why saline elicited peaks of cortisol at 0 minute post-injection in a female (#138724) and nearly all swabs post-ACTH for one male (#139131). However, the overall response to the saline was lower (approximately 1-fold) than the response to the ACTH (about 3-fold) in female Wyoming toads, while males were similar (about 2-fold with saline compared to <1-fold with ACTH). Other species have responded to the control saline injection during an ACTH challenge, including the green treefrog, red-spotted newt (Santymire *et al.*, 2018) and edible bullfrog (Scheun *et al.*, 2019). However, the American toad had no response to the saline injection (Santymire *et al.*, 2018). Similarly, in ACTH challenges with waterborne corticosterone in aquatic salamanders, Gabor *et al.* (2016) and Nagel *et al.* (2019) found no significant difference in waterborne corticosterone in ACTH and saline treatments, but Gabor *et al.* (2016) found that ACTH-treatment salamanders exhibited higher GCs than non-injected control salamanders.

### Faecal GC metabolites

When validating the use of FGM analysis, we only had two Wyoming toads (one male, one female) that provided uncontaminated faecal samples before and after the ACTH injection. This limited our study, and future studies should include more individuals. However, in the current study with six Wyoming toads (two ACTH; four saline) that provided post-injection samples, we did confirm that the Wyoming toads responded to the ACTH challenge with significant increases in FGM the week after ACTH injection. We found no change in the saline injected toads. Similarly, in harlequin frogs, an ACTH challenge resulted in 4- to 5-fold higher increases in faecal cortisol metabolites 3 to 4 days post-injection (Cikanek *et al.*, 2014).

Because of the lag time between dermal hormones and the time it takes to metabolize these steroid hormones, our FGM and dermal cortisol results are not directly comparable. However, we compared the overall FGM and dermal cortisol profiles and found that the ACTH male (#139008) had lower FGM and lower dermal cortisol than most of the other toads. Although we did not observe an increase in GC via swabs, the male had two significant FGM peaks post-ACTH. The female (#139170) had four peaks in dermal cortisol, one of which was the largest peak (~840 pg/ml swab) observed in all toads sampled. This female also had the highest FGM values. Although these samples are at different time points (soonest peak post-ACTH was Day 1 in female and Day 2 in male), it is interesting to observe a possible correlation between overall FGM and dermal cortisol value.

### Reintroduced Wyoming toads

Unfortunately, Wyoming toads were only found at three of the six reintroduction sites. At the time of the August surveys, ponds had dried at Red Buttes, Outrider and McGee. Limited water was present in intermittent shallow creeks and ditches, but no toads were detected in these areas. Water was still present in large ponds at Mortenson, Buford and Lindzey during the VES, and most toads encountered were in or adjacent to the ponds on saturated ground. This seasonal effect on hydrology may be influencing the ability to locate reintroduced Wyoming toads and their GC production.

We found that dermal cortisol was similar among reintroduced males, females and unsexed juveniles. Homan *et al.* (2003) found that although GCs differed between male and female spotted salamanders, they did not differ year-round. However, it has been shown that males and females can respond differently to behavioural and physiological stressors (reviewed in Touma and Palme, 2005; Fijian ground frogs, Narayan *et al.*, 2010). The reintroduction sites where Wyoming toads were found did not produce site-level differences in dermal cortisol, but these three sites generally had similar moisture regimes and generally greater abundance and detection rates than Outrider, Red Buttes and McGee, where no toads were encountered during August surveys. Because of the lack of toads in the three lower detection rate sites, our ability to address reintroduction site differences in dermal cortisol was limited. Future surveys conducted earlier in the season or in a year when water is still present on the landscape in Outrider, Red Buttes and McGee, may elucidate site differences in GCs. However, time of year may also affect cortisol production. We found that dermal cortisol in reintroduced toads was significantly higher in August compared with June. Weather did not vary greatly between sampling periods, but the drier conditions in August could be a possible stressor for the toads, along with lower overall energetic reserves following breeding. It is possible that our evaluation of reintroduction sites used too coarse of a filter and that more detailed habitat data could reveal relevant differences in GC levels.

Like our dermal cortisol results, *Bd* prevalence has been found to consistently increase over the summer (June to August) in Wyoming toads, both in positive cases and zoospore loads (US Fish and Wildlife Service, 2018). We found that two of six toads (33%) were *Bd* + in June compared with 21 of 27 (78%) *Bd* + toads in August. *Bd* has been implicated in mass die-offs of naïve amphibian populations (Crawford *et al.*, 2012; Olson *et al.*, 2013). Previous research has investigated the relationship between *Bd* and GC production in tadpoles and juveniles and found, most commonly, a positive relationship (six of ten; summarized in Hammond *et al.*, 2020). For example, two species of *Alytes* tadpoles had higher urinary corticosterone when *Bd* + (Gabor *et al.*, 2013). However, previous research had not found a direct relationship between exogenous corticosterone and *Bd* susceptibility in the larvae stage of *A. boreas*, *R. cascadae* and *Lithobates catesbeianus* and post-metamorphic *R. cascadae* (Searle *et al.*, 2014). In juvenile American toads, exogenous corticosterone enhanced resistance, but not their tolerance to *Bd* (Murone *et al.*, 2016). In adults, previous studies have found a positive relationship between plasma (*Plethodon shermanii*, Fonner *et al.*, 2017; *Litoria caerulea*, Peterson *et al.*, 2013) and urinary (Stoney Creek frog, Kindermann *et al.*, 2012, 2017) corticosterone and *Bd* status (Hammond *et al.*, 2020). Here, we did not find any significant relationship between *Bd* status and dermal cortisol concentrations in reintroduced Wyoming toads. However, in August, we did observe increased dermal cortisol levels along with nearly 80% of Wyoming toads positive with *Bd*, which may indicate an overall response by toads in the study sites to the greater zoospore load in the environment. An increased sample size or repeat sampling of the same individual over multiple months may prove to be more informative in regards to the effects of *Bd* on individual stress physiology.

### Comparing captive and reintroduced Wyoming toads

When comparing reintroduced and captive Wyoming toads, we found that reintroduced Wyoming toads had 3-fold higher dermal cortisol than captive individuals. Based on previous research, we knew that capture methods could increase dermal GC production (Santymire *et al.*, 2018), so we swabbed the toads immediately upon capture. Therefore, we suspect that the stressors causing elevated dermal cortisol in the reintroduced population originated in the environment and could have been a function of the time of year or other factors such as predation pressure, water chemistry, moisture, temperature, food availability or *Bd* status (Chambers *et al.*, 2013; Burraco and Gomez-Mestre, 2016, reviewed by Narayan *et al.*, 2019; Walls and Gabor, 2019). Previous research has found similar dermal GC values between wild and wild-caught but previously captive amphibians including the Northern leopard frog (*L. pipiens*) and American toad (Santymire *et al.*, 2018). Narayan *et al.* (2010) found that captive male Fijian ground frogs had lower urinary corticosterone than wild males when not considering seasonal effects; however, captive males and females had similar levels.

Here, we found that ACTH stimulated the HPI axis, resulting in detectable cortisol increases in both dermal swabs and faecal samples. Although not practical for fieldwork, we did validate the use of FGM analysis, which could be a useful tool for captive toad research. However, dermal cortisol analysis could be useful for both captive and wild research.

Although there was not a significant sex difference, stress responses varied among individuals. Additionally, dermal cortisol was influenced by time of year with reintroduced toads having higher dermal cortisol in August than toads sampled in June. We caution researchers that it important to standardize swabbing methods between individual collectors to reduce variation. Here, we swabbed toads up and back three times (totaling six passes with the swab) spanning 1 inch. Similar to *Bd* swab collection (Simpkins *et al.*, 2014), collector effects may influence sample collection, if the swabbing method varies between individuals, more or less hormone may be collected, thus producing varying results that are not biologically relevant to the animal. Also, it is important that the patch of skin where the sample will be collected from on the dorsum of an amphibian is dry because any added moisture to the swab could add volume to the 1 mL of ethanol, thus resulting in some variation between individuals. However, this swabbing method has been used in fully aquatic species including axolotl, newts, mudpuppies (Santymire *et al.*, 2018) and even three cephalopod species where individuals were swabbed underwater (Chancellor *et al.*, 2021). Here, the toads were dry and did not foam or urinate during collection; thus, the variation was most likely a reflection of biological differences among individuals.

The next steps should include using high pressure liquid chromatography to determine the biochemical nature of these steroids to ensure that we are measuring dermal cortisol. Additionally, it will be important to determine the impact of these GCs on the individual (Dickens and Romero, 2013; Madliger *et al.*, 2018). Allostatic load, which is the collective damage to the individual’s physical being due to repeated efforts to return to homeostasis (reviewed in Juster *et al.*, 2010), can result in a dysfunctional HPA (HPI in amphibians) axis and make responses difficult to interpret (Romero *et al.*, 2009; Sapolsky *et al.*, 2000). Therefore, lower GCs may not necessarily mean that the individual is less stressed. Future studies should compare GCs to fitness measures, such as survival, health and reproduction. Both the dermal swabs and faecal hormonal metabolite analyses could be used to measure reproductive steroid hormones. These methods can increase our understanding of Wyoming toad reproductive biology and not only provide information on the impact of GC production but could also assist with captive breeding and understanding the relationship between GC production and reproductive success. In the field, dermal hormone analysis can be used to help better understand how environmental conditions are affecting Wyoming toad recovery.

## Supplementary Material

supp_coab093
