# Supplementary material for: Using dermal glucocorticoids to determine the effects of disease and environment on the critically endangered Wyoming toad
Source: Conserv Physiol. 2021 Dec 23;9(1):coab093. doi: 10.1093/conphys/coab093 (PMC8849142; doi:10.1093/conphys/coab093)
Supplement: supp_coab093 [file supp_coab093.docx]

**B**

D

A

**A**

Supplementary Figure 1. Parallelism between dermal cortisol (A), dermal corticosterone (B), fecal cortisol metabolites (C) and fecal corticosterone metabolites (D) and the enzyme immunoassay’s standards.

**A**

Supplementary Figure 2. Percent recovery results for dermal cortisol (A), dermal corticosterone (B), fecal cortisol metabolites (C) and fecal corticosterone metabolites (D) and the enzyme immunoassay’s standards.

Supplementary Figure 3. Dermal cortisol and corticosterone (pg/ml swab) results over time in captive Wyoming toads after receiving a saline injection: A. Male #139292; B. Female #138724; C. Female #138695; and D. Male #139131.

Supplementary Figure 4. Dermal cortisol and corticosterone (pg/ml swab) results over time in captive Wyoming toads after receiving an adrenocorticotropic (ACTH) injection: A. Female #139221; B. Male #139008; C. Female #139170; and D. Male #138661.

A

B

Supplementary Figure 5. Fecal cortisol and corticosterone metabolite (ng/g dry feces) results over time in captive Wyoming toads after receiving: A. an adrenocorticotropic (ACTH) injection or B. a saline injection.
